# Topological Considerations in Biomolecular Condensation

**DOI:** 10.3390/biom13010151

**Published:** 2023-01-11

**Authors:** Debapriya Das, Ashok A. Deniz

**Affiliations:** Department of Integrative Structural and Computational Biology, The Scripps Research Institute, 10550 N. Torrey Pines Rd., La Jolla, CA 92037, USA

**Keywords:** intrinsically disordered proteins, polymer physics, percolation, entanglement, RNA, topology, polymer rheology, biomolecular condensates

## Abstract

Biomolecular condensation and phase separation are increasingly understood to play crucial roles in cellular compartmentalization and spatiotemporal regulation of cell machinery implicated in function and pathology. A key aspect of current research is to gain insight into the underlying physical mechanisms of these processes. Accordingly, concepts of soft matter and polymer physics, the thermodynamics of mixing, and material science have been utilized for understanding condensation mechanisms of multivalent macromolecules resulting in viscoelastic mesoscopic supramolecular assemblies. Here, we focus on two topological concepts that have recently been providing key mechanistic understanding in the field. First, we will discuss how percolation provides a network-topology-related framework that offers an interesting paradigm to understand the complex networking of dense ‘connected’ condensate structures and, therefore, their phase behavior. Second, we will discuss the idea of entanglement as another topological concept that has deep roots in polymer physics and important implications for biomolecular condensates. We will first review some historical developments and fundamentals of these concepts, then we will discuss current advancements and recent examples. Our discussion ends with a few open questions and the challenges to address them, hinting at unveiling fresh possibilities for the modification of existing knowledge as well as the development of new concepts relevant to condensate science.

## 1. Introduction

**Figure 1:**
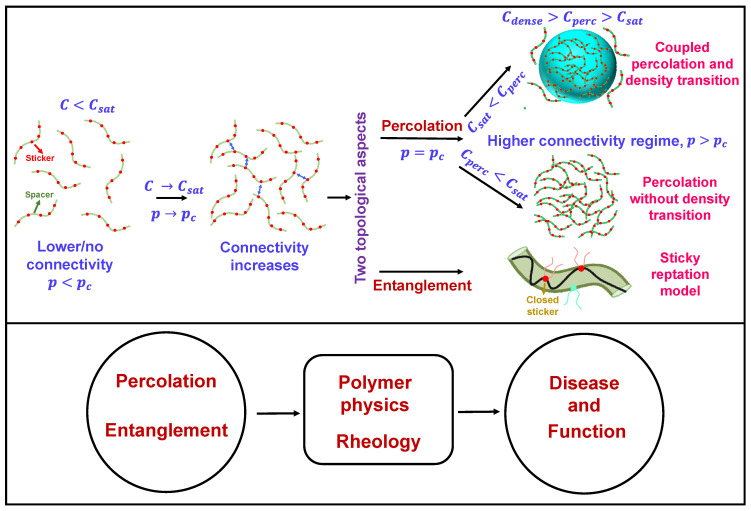
General overview of two topological concepts discussed here, namely percolation and entanglement in the context of biomolecular condensation. In the upper panel (on left side) the red circles describe the stickers and the green circles constitute the spacer. The blue two-sided arrows describe sticker-sticker interactions. On right side, the droplet has been shown by a light blue sphere with color coded intersticker interactions. In the ‘sticky reptation model’ the polymer chain confined in the tube (olive) is shown by a black strand. The red circles with strand describe the ‘closed stickers’ and cyan circles describes the next available polymer (sticker) chain for reptation depending upon the chain diffusion pattern. This concept has been discussed in detail in later sections of this review.

Biomolecular condensation via phase separation (PS) of proteins and nucleic acids is believed to play a pivotal role in cellular compartmentalization and spatiotemporal regulation of cellular biochemistry, which are associated with an array of essential biological functions and debilitating neurodegenerative dysfunctions [1,2,3,4,5,6,7,8,9,10,11]. Intracellular biomolecular phase-separated structures, also known as membrane-less organelles, are viscoelastic dynamic mesoscopic supramolecular assemblies with various cluster size distributions within the biological milieu [5,12,13,14,15]. A key driving force of PS is the multivalency of different interaction domains comprising the condensates [5,12,16], resulting in the formation of dense noncovalent networks/crosslinks within the system. The traditional mean-field Flory–Huggins theory of homopolymer in solution illustrates the thermodynamics and the physical understanding of the phase transition [17,18,19,20,21,22]. The derived Flory–Huggins interaction parameter (χ) allows us to quantify the intricate balance between chain–chain, chain–solvent and solvent–solvent interactions, thereby dictating the phase separation propensity of the system, depending on the solvent quality. Although the mean-field model has been widely utilized to understand the characteristics of phase behavior, it may not offer a good approximation to understand the complex nature of phase transitions involving larger macromolecules such as proteins and RNA, which are believed to be major drivers of intracellular bimolecular condensation [5,23,24,25]. The phase separation of these molecules possessing multiple intrinsically disordered regions (IDRs) and/or folded domains involves a mosaic of sequence-dependent, structurally, and conformationally heterogeneous dynamic multivalent interactions. In order to gain insight into the mechanistic understanding of these processes, the theory of linear or branched associative polymers has been proposed using stickers-and-spacers network architecture [5,16,26,27,28,29,30,31]. In this framework, depending on the system-specific sticker–sticker interaction, the physical crosslinking amongst them leads to two types of transitions: (1) phase separation or density transition above a critical protein concentration $\left(C_{sat}\right),$ forming a polymer-rich dense phase $\left(C_{dense}\right)$ cohabiting with a polymer-deficient dispersed phase $\left(C_{dil}\right)$; (2) percolation, which is a topology-related geometric transition that depends on the connectivity probability and leads to the formation of system-spanning clusters [23,28,29,31,32] (Figure 1). Phase separation and percolation may be coupled or decoupled depending on the system and other specific parameters. Percolation theories, which were developed in early work in connection with graph and network theories, can be employed to better understand the intricate details of biomolecular condensation [23,32,33,34,35,36,37]. Another captivating topological concept is polymer entanglement, which has recently been implicated in biomolecular condensates and their rheological properties [38,39,40] (Figure 1). In this review, we discuss the underlying physical origins of percolation and polymer entanglement and their relevance in the context of PS and gelation of associative biopolymers from both a historical and scientific standpoint. We note that the application of these exciting concepts to biomolecular condensates is at a relatively early stage, and we discuss current limitations and future directions in the final section of this article. We envision that the application and invocation of topology-network-related theories may shed light on different aspects of biomolecular condensation within the cellular milieu.

## 2. Percolation Physics

### 2.1. Percolation Physics: A Historical and Scientific Overview

**Figure 2:**
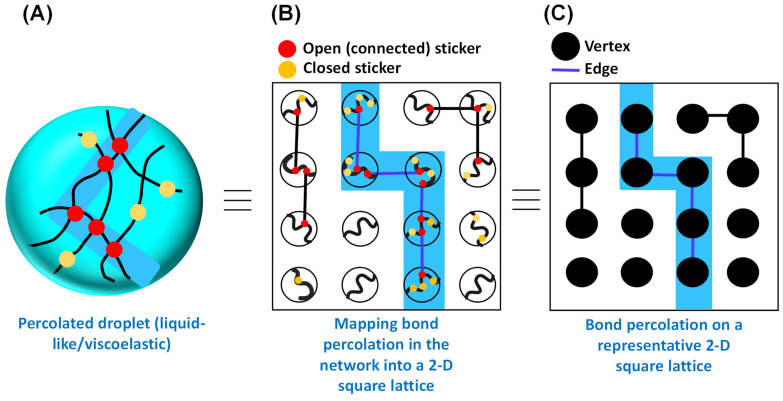
Mapping the bond percolation of a representative 2-D square lattice onto the physical crosslinks in the percolated network within the phase separating biomacromolecules. (**A**) Schematic representation of physical crosslinking formed by sticker–sticker interactions within the percolated droplet. Polymer chains are shown in black. Red circles define the open (connected) stickers, and yellow circles define the closed stickers. The percolating cluster (open path) is shown by the light blue shade. (**B**) Conceptualization of bond percolation in the context of percolated droplet. (**C**) Bond percolation on a representative 2-D square lattice, as proposed by Broadbent and Hammersley in the context of an arbitrary linear graph with vertices and edges. The color code remains the same as (**A**).

***Theoretical foundation and different models.*** For a simple visualization, we can consider percolation as a simplified probabilistic model for a porous rock in which the interior of the rock is depicted to be a random maze through which fluid can flow. In this context, an important question to ask is which part of the rock will become wet after being submerged in the fluid. Mathematically, the porous material can be depicted by a random graph with vertices and edges, as first described by Broadbent and Hammersley in the 1950s. They first introduced the term ‘percolation’ in the context of their novel mathematical problems concerning the flow of a liquid through a random maze, hence the name ‘percolation’ [35,36]. A percolation model is defined as a collection of points with a spatial distribution in which certain pairs are shown to be connected. Depending on the model, the nature of connectedness is random, which suggests that each of these connected structures has a certain statistical probability of occurring. We focus here on the bond percolation model, which can be most intuitively mapped with biomolecular condensates (Figure 2), and note the existence of other models such as site, continuous, and hybrid percolation models. Before we proceed further, to motivate the following discussion of percolation theory through a more concrete link between protein/RNA condensates/networks and lattice percolation models, we point to Figure 2. Here, the reader can see a simple conceptual mapping of a reversibly crosslinked condensate-forming macromolecular system as commonly depicted in the field (Figure 2A; e.g., of disordered proteins or RNA) and bond percolation on a 2-D square lattice (Figure 2C), via an intermediate map of Figure 2B. In a related point, we also would like to emphasize that although fluid flow was used to conceptually introduce percolation models and its historical background, the relevant concept for biomolecular condensates is percolation through bonds, as depicted in Figure 2A,B.

Broadbent and Hammersley originally introduced the bond percolation model in the context of graph theory [36]. According to this model, in an arbitrary linear graph, a certain pair of vertices or points forming an edge in the graph are connected with probability p independent of the connectivity of other pairs, considering no edge formation between the pairs without linkages (Figure 2C) [35,40,41]. Figure 2C shows a general model of bond percolation on a two-dimensional square lattice in which the points of the model represent the lattice sites and each closest neighboring pair is linked with probability p. The points possess fixed locations, and the linkage (bonds) formation can occur randomly, and the properties of the model are determined by the topology of the network. Therefore, in the case of a square lattice, in the bond percolation model, lattice edges are the relevant entities, and the substance (fluid) seeps through the adjacent bonds. This idea may directly be mapped onto the concept of passage of liquid through the open path (physical crosslinks) formed by the sticker–sticker interaction, as depicted by Figure 2A,B. In a broader sense, if we recall the concept of percolation through a porous rock, the open edges allow the fluid to pass through, with the closed edges blocking the percolation. 

**Figure 3:**
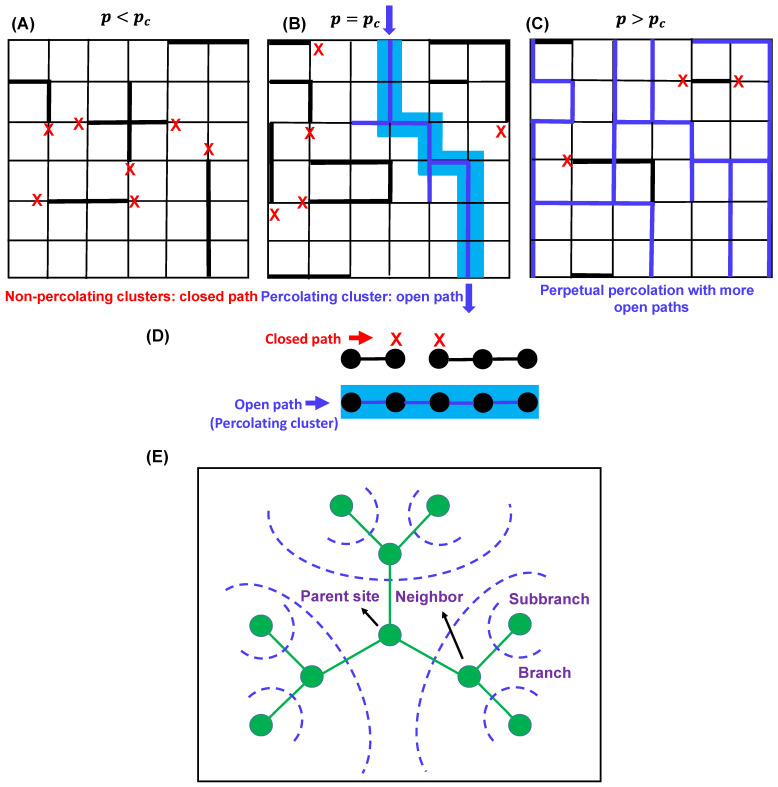
Three lattices with different percolation thresholds ($p_{c}$) conditions: (**A**) $p<p_{c}$, (**B**) $p=p_{c}$, and (**C**). Percolation occurs when $p=p_{c}$. In (**B**), the bond connectivity is shown by blue lines, and the percolating cluster is shown in light blue shade. Red crosses describe closed paths. In (**C**), the bond connectivity is shown by blue lines, red crosses describe closed paths, and the clusters are not shown for simplicity. (**D**) Bond percolation in one dimension. The vertices are shown by black circles, and the edges (bond connectivity) are shown by black lines. Red cross describes the closed paths, and blue line describes the open paths for percolation. Percolating cluster is shown by light blue shade. (**E**) Typical sketch of a Bethe lattice with coordination number 3. Parent site, branches, and subbranches are shown by olive circles. The blue dashed lines depict possible directions of branching.

In percolation theory, the phrase ‘percolation threshold’, denoted as $p_{c}$ defines the (connectivity) probability that ‘marks the birth’ of an infinitely connected cluster. In other words, it measures how likely a particular point is to be a part of an infinite cluster [35,40,42]. In the context of fluids, this is the probability that a fluid introduced at the point will percolate away through the ‘open paths’ within the system perpetually (Figure 3A–C). The cluster size increases as the number of linkages increases, and at a given critical density of linkages, it crosses the percolation threshold, and the extent of cluster size increment may become infinite, at which point the system is considered to be percolating. When $p<p_{c}$, the system lacks infinitely connected components, whereas above $p_{c}$, the system will possess at least one such cluster (Figure 3A–C). Therefore, $p_{c}$ marks the critical transition point from a low (local) to a dense (global) connectivity regime. 

Next, we will briefly discuss the analytical treatment of the percolation problem in one dimension as a simple example. 

***A simple example–percolation problem in one dimension*** Consider a one-dimensional lattice with an infinite number of equally spaced nodes [41] (Figure 3D). The probability of bonds between adjacent sites is denoted as p (open) giving rise to a (1−p) probability of no bond. The question is as follows: what is the critical value of the percolation threshold $p_{c}$ or the bond probability at which an infinite cluster arrives for the first time? 

Let us denote ∏(p,L) as the probability of percolation at p for a lattice of linear size L. Therefore, in line with our previous discussion, two scenarios can emerge, which are as follows,

$$\underset{L\to \infty }{\lim}\prod \left(p,L\right)=\{\begin{array}{c} 0\;for\;p<p_{c}\\ 1\;for\;p\geq p_{c} \end{array}$$

In the case of the 1-D finite lattice of size L, all nodes are occupied with probability $\prod \left(p,L\right)=p^{L-1}$, as the events of occupation are independent of each other, and it gives rise to the following,

$$\underset{L\to \infty }{\lim}\begin{array}{c} \prod \left(p,L\right)=\underset{L\to \infty }{\lim}p^{L-1}=\{\begin{array}{c} 0\;for\;p<1\\ 1\;for\;p=1 \end{array} \end{array}$$

which implies $p_{c}=1$. 

This solution is in line with the idea that for a 1-D lattice, percolating cluster formation can occur with all adjacent sites forming bonds only when $p_{c}=1$, because even a single ‘no bond’ situation would block a cluster to percolate through the lattice [41]. The one-dimensional percolation problem demonstrates several traits present in higher-dimensional systems, and it furnishes a clear starting point to understand the fields of scaling concepts, phase transition, renormalization group theories, and so forth [35,41]. Therefore, the mathematical treatment of a simple one-dimensional problem may aid in delving deeper into the more complex percolation problems in higher dimensions. 

***Percolation: a topology-driven phenomenon*** A percolation process describes the transition from an initial structure comprising a set of isolated objects to a system with an inter-connected structure as a function of increasing density. In any geometric structure or field, the presence of points of two opposing edges or planes belonging to the same connected component indicates that the system or structure has the potential to undergo percolation. As the connectivity increases, the intrusion of the fluid approaches completion. Therefore, it is a topology-driven phenomenon, as the addition of more ‘connectivity’ to the structure modifies the underlying topology [42,43,44,45,46,47]. We note that the percolation threshold depends on different parameters of the model, including the lattice type in different dimensions. For instance, for the bond percolation model, *p_c_* = 1 for a one-dimensional lattice, as noted above, whereas *p_c_* = 0.5 for a two-dimensional square lattice [40,44,45]. In general, the percolation threshold decreases as the coordination number of the lattice increases in each dimension. Increasing functionality would progressively introduce more complexity in the percolating behavior of the system [40]. Next, we will discuss the relevance of percolation transitions in the context of gelation and phase separation.

### 2.2. Percolation Approach: Gelation and Phase Separation

***General concepts of gelation*** To put it simply, a polymerization process is initiated starting with a liquid containing monomers with higher reaction functionality (f). This eventually results in a transition from liquid to solid (gel). This idea was first described by the classical model of gelation developed by Flory and Stockmayer [20,48]. This is a mean-field approach based on several assumptions, such as not considering the possibility of intramolecular linkage formation (cyclization) and treating all unreacted functional groups as equally active at any stage of the reaction. According to their theory, gelation behavior is observed in systems with higher functionality and with a possibility of unrestricted growth capability resulting in the formation of indefinitely large three-dimensional molecules. Flory’s theory furnishes a general ‘critical’ value $\alpha_{c}$ for the formation of this infinitely large network, which is as follows,

(1) $$\alpha_{c}=\frac{1}{f-1}$$

where f is the functionality of the branch units and α is the probability of the chain branching as opposed to chain termination, depending on various parameters such as the ratios of the reactants and the reaction capability of the functional groups. Approximately, we can consider the branching probability α to be equivalent (not necessarily equal) to the extent of the reaction, related to p [49]. Concisely, when the degree of branching and crosslinking events exceeds a critical value, three-dimensional polymerization causes gelation due to network formation to an indefinite extent. Following that, we will direct our efforts toward understanding gelation in light of the percolation approach.

***Gelation: a bond percolation transition*** Flory–Stockmayer theory is the cornerstone of percolation models undergoing a transition from a state of local connectedness to one in which the connections extend indefinitely. From this perspective, gelation can be described as the connectivity transition from sol to gel that can be modeled by bond percolation theory, such that all sites of the lattice are occupied by monomers [5,16,29,33,40]. The extent of the networking increases as a function of crosslinking from 0 to 1. When the system reaches the percolation threshold or the gel point, it undergoes a connectivity transition [48]. In this case, we must consider two scenarios: (1) when the system is slightly below $p_{c}$, it is a polydisperse mixture of branched polymers; (2) when the system is slightly above $p_{c}$, the network is not fully developed, and only one structure seeps (percolates) through the entire system, as discussed in depth by Rubinstein and Colby [40]. The sol fraction $\left(P_{sol}\right)$ is the fraction of monomers that are part of the finite-size polymers, and the gel fraction $\left(P_{gel}\right)$ is the fraction of all the monomers that belong to the gel network. From these ideas, we can depict the following conditions as shown by Equation (2a–c) [40].

(2a) $$P_{sol}+P_{gel}=1$$

(2b) $$P_{sol}=1,\;P_{gel}=0,\;p\leq p_{c}$$

(2c) $$P_{sol}<1,\;P_{gel}>0,\;p>p_{c}$$

As previously discussed, percolation effects are dependent on the lattice type and functionality; in this context, it is worthwhile to discuss the mean-field gelation model, which corresponds to bond percolation on a Bethe lattice (Figure 3E) [40,41]. The simplest random bond percolation model on a Bethe lattice directly considers the functionality of the monomers by adopting this functionality for the lattice which, unlike a simple cubic lattice model, assumes the absence of any intermolecular crosslinking and is convenient for analytical treatment of the model. Consistent with Flory’s equation and the analytical treatment of the one-dimensional percolation problem, the critical occupation probability or the gel point for the bond percolation model of an ‘infinite-dimensional’ Bethe lattice is given by the following equation [40],

(3) $$p_{c}=\frac{1}{f-1}$$

where each site possesses f number of neighboring sites; therefore, each branch gives rise to f−1 subbranches. Here, below the gel point, only finite-size branched clusters exist, and above the gel point, in addition to that, at least one infinite polymer exists. Figure 3E shows a sketch of a typical Bethe lattice with functionality (coordination number) 3, with a large number of independent branching probabilities (p) starting from the parent site. Interestingly, a distinct feature of percolation on a Bethe lattice is the presence of a significant number of infinite polymers in the system just above the gel point, as opposed to the regular lattice in which only one infinite polymer exists above the gel point [40,41]. Next, we will shed light on understanding the interplay between percolation and sol–gel transitions in the context of biomolecular phase condensation.

***Interplay between percolation and biomolecular condensation*** Macromolecular systems such as proteins can be considered in the framework of sticker–spacer-based associative polymer models, founded on an equilibrium theory originally developed by Semenov, Dobrynin, and Rubinstein in the context of reversible network formations in solutions of polymers with many associating groups, namely stickers (which are generally the functional monomeric units, charged moiety, or hydrophobic group) per chain [5,16,27,28,29,31]. As opposed to the mean-field assumption, this model takes into account the specific pairwise attractive interaction between stickers. The spacers are considered to be noninteracting and, thus, behave as ideal chains that are interspersed between stickers, without much influence in the formation of physical crosslinks but contribute toward the excluded volume effects implicated in the overall association of the polymers [5,16,50]. The reversible intersticker interactions give rise to two physical events: (1) intermolecular clustering and gelation transition, and (2) phase separation as a function of increasing intersticker interaction potential. The phase behavior of associative polymers is theoretically based on the classical gelation theory proposed by Flory and Stockmayer and the theory of polymer solutions developed by Flory [21,48,49]. Because of the reversible nature of the crosslink formation, a specific polymer chain can reversibly be a part of a sol phase (finite cluster) or gel phase (infinite cluster along with finite clusters), as opposed to chemical gelation in which the bonds are not reversible. Associative polymer models with sticker–spacer paradigms offer a useful platform for elucidating the physical attributes of biomolecular condensation.

During biomolecular condensation, a percolation transition occurs when protein and/or nucleic acid molecules (such as RNA) are topologically connected into a system such that the connectivity percolates throughout the system, giving rise to a droplet spanning network matrix (Figure 2) [51]. The critical concentration $\left(C_{perc}\right)$ for connectivity transition or the percolation threshold depends on the types and valence behavior of the stickers, sticker–sticker interaction potential, and spacer-mediated solvation effects [5,33]. When $C_{perc}<C_{sat}$, a percolation transition can occur without phase separation, forming an ‘infinite polymer’ or gel depending on the degree of reversible crosslink formation. Interestingly, when $C_{sat}<C_{perc}<C_{dense}$, the polymer solution should be able to undergo phase separation coupled with percolation (PSCP), leading to the development of a droplet-spanning percolated matrix [16,24,51]. As theorized by Semenov and Rubinstein [28] and also discussed by Choi et al. [5], for a system comprising associative polymers in a solvent with *n* number of self-interacting stickers, the percolation threshold of the system is given by the following equation,

(4) $$C_{perc}=\frac{1}{\lambda n^{2}}$$

Here, the stickers are considered to be phantom chains. n= apparent valence of stickers, and $\lambda =v_{b}e^{-\left(\frac{\varepsilon }{k_{B}T}\right)}$, where $v_{b}=$ intersticker crosslinking volume, ε= effective interaction energy between the stickers (𝜀 ≤ 0), $k_{B}=$ Boltzmann constant, and T= temperature of the system. 

Graph-based Monte Carlo simulations carried out by Choi et al. described the concept of phase-separation-aided bond percolation (PSBP) using the sticker–spacer framework of associative polymers [33]. The mean-field model ignores the effect of growing network connectivity and forming clusters below $p_{c}$. These clusters form as a result of pairwise sticker interactions between different polymers involved in physical crosslinking, as well as the bond cooperativity effect, which deals with the effective intersticker interaction influenced by the previously generated interaction and, thus, can alter the percolation behavior of the system. Overall, these concepts are in line with the classical gelation model which was pictured by Flory and Stockmayer and the Flory–Huggins model of polymer solutions, with the correction for the mean-field approach. In light of this, associative polymer theories with the inclusion of the percolation approach can describe sticker–spacer-based macromolecular phase separation-assisted bond percolation (PSBP).

### 2.3. Current Implementation and Biological Implications

The concept of percolation effects has recently been applied to the area of biomolecular condensates and assemblies, which has helped us to delve deeper into the mechanistic characteristics of PS, liquid–solid transition (gelation), percolation effects on phase transitions, nano- and mesoscale cluster formation, and so forth. In 2012, Li et al. reported on the phase behavior of systems in which phase transitions are fueled by multivalent interactions between poly-SH3 and proline-rich (poly-PRM) molecules [24]. They showed that these interactions are driven by the unique association ability of the SH3*_n_*-PRM*_n_* molecules, implying a valence-specific percolation threshold for phase separation to occur. Further evidence indicated that macroscopic phase separation is thermodynamically coupled to a sol–gel transition within the droplet state, which is an example of PSCP that eventually leads to the formation of gel, as previously discussed. They noted that, as well as being generic features of multivalent macromolecular biological systems, these phenomena with sharp phase transitions could impact the cellular signaling pathways or contribute to the structural and functional ability of cellular components [24]. By adopting this synthetic system, SH3*_n_*-PRM*_n_*, Harmon et al. performed Monte Carlo simulations using a coarse-grained lattice model with different valencies to demonstrate the effect of intrinsically disordered linkers, namely Flory random coil linkers (FRC) and self-avoiding random coil linkers (SARC), and their effective solvation volume on gelation with and without phase separation [16,50]. Their studies revealed that at bulk concentrations below the Flory–Stockmayer limit, gelation with phase separation results in positive global cooperativity and leads to the generation of a percolated network. On the other hand, gelation without phase separation is preferred in systems with zero or negative global cooperativity, and the transition takes place at or above the Flory–Stockmayer limit. The authors speculated that cell-signaling regulation is primarily modulated by gelation-driven phase separation of multivalent proteins, with specific interaction motifs or linear domains leading to the formation of percolated networks based on the theory of associative polymers. A few years ago, Franzmann et al. investigated the pH-regulated PS of Sup35 and subsequent solidification into a porous mesh-like polymer network or crosslinked protein gel driven by the intrinsically disordered prion domain [52]. This phenomenon is consistent with the idea of gelation driven by phase separation, but the complex mechanism underlying the formation of crosslinked meshwork remains elusive. We can speculate that the percolation transition might play an important role in the conversion from liquid-like droplets to reversible permeable gel or gel-like condensates. This intracellular phase separation coupled with gelation offers a beneficial way for cells to respond to sudden environmental stress.

Interestingly, recent work by Kar et al. has shown that subsaturated solutions of FET family proteins contain a variety of nanoscale clusters, even though micron-scale phase separation is not seen in solutions below an effective $C_{sat}$ [32]. In general, a subsaturated solution is expected to contain mostly dispersed monomers, along with very few small clusters at a time, and the phase separation is governed by the unique Flory interaction parameter χ [21]. Thus, interestingly, their results do not reconcile with this conventional notion. The authors discuss how the results are instead consistent with the presence of multiple relevant energy scales in the system, including one that relates to percolation clustering. The generation of smaller networks below the gel point can be understood from the viewpoint of percolation theory, in which below the gel point, the connectivity is low, thereby forming percolation clusters (termed pre-percolation clusters in the work of Kar et al.). Above the gel point, percolation commences, and the size distribution of the clusters increases as a function of increasing connectivity. The authors also report the results of simulations that also are consistent with a model involving percolation clustering. Notably, Li et al. had previously reported the presence of mesoscale percolation clusters below $C_{sat}$ during PRM-SH3_5_ titrations characterized by dynamic light scattering (DLS) and small-angle X-ray scattering (SAXS) and connected the observations to percolation [24]. 

Recently, Cho et al. demonstrated that many RNA-binding proteins form clusters (potentially similar to percolation clusters, as speculated by Kar et al.) under biologically relevant unstressed conditions, which could eventually drive the onset of phase separation under stressed conditions [53]. Recent work by Zhao et al. featured the generation of supramolecular clusters in the subsaturated solution of SARS-CoV-2 N-protein prior to the formation of phase-separated droplets [54]. Seim et al. demonstrated the intricate interplay between homotypic and heterotypic interactions, which drives the phase separation coupled to percolation in their fungal protein Whi3 and RNA system [51]. Interestingly they also observed the presence of heterogeneous distribution of percolation clusters in the sol (dilute) phase cohabiting with the dense phase, which is the embodiment of PSCP. Previously, Vorontsova et al. showed the presence of mesoscopic clusters with low occurrence in the subsaturated solutions of lysozyme [55]. In that case, the protein-rich clusters of a definite size, independent of the protein concentration variation, indicate microphase separation as opposed to percolation-type clustering and were suggested to be the precursors to the formation of protein aggregates, amyloid fibrils, and crystals [55,56,57,58]. Another example is work by Frey et al., which showed that phenylalanine-glycine (FG) repeats of nuclear pore proteins undergo sol–gel transition via noncovalent reversible crosslinking, which is critical for viability in yeast [59]. 

In computational work, Ranganathan et al. demonstrated that for a multivalent sticker–spacer protein complex, there is a dynamic interplay between two competing processes: (1) protein–protein interactions limited by diffusion and (2) loss of available valency within the smaller clusters engendering kinetically trapped metastable multi-droplet states [60]. They observed a slowdown of the dynamics of the condensed phase in the regime favoring large clusters, which may result in functional loss. This is an interesting phenomenon in which the metastability of the dynamic cluster controls the progress (kinetics) of the phase transition reaction, and percolation behavior might play an important role in the increasing network connectivity event. Overall, percolation is a networking transition governed by specific multivalent interactions which may (PSCP) or may not result in phase separation. The PSCP paradigm is pertinent to defining the phase behavior of multivalent biomolecules with the sticker–spacer framework and engenders sequence-, chemistry-, and topology-specific clusters, which results in network fluids, as opposed to with pure LLPS [6]. In the case of percolation without phase separation, a system-spanning percolated network is formed. All these physical states may be functionally relevant on the mesoscale, depending on the structural and dynamical properties of the condensate-/system-spanning physical crosslink engendered from the sticker–sticker network. Nevertheless, all of these intriguing observations point directly to the possibility that percolation coupled or decoupled with phase separation may play a vital role in biology and may fill the gap between micro- and macroscopic phase separation in cellular biochemistry. 

## 3. Entanglement Effects

**Figure 4:**
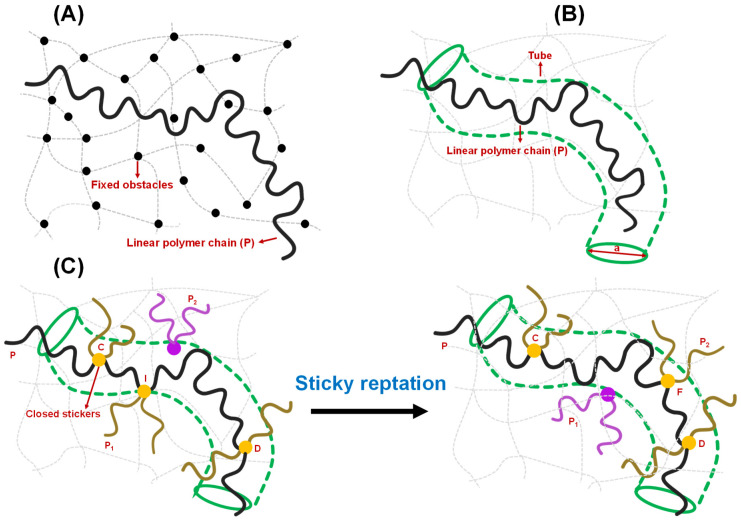
Different models of polymer entanglement. (**A**) Conceptualization of reptation of a polymer chain (P) in the presence of fixed obstacles, as theorized by de Gennes. The chain can freely move between the fixed obstacles but is not allowed to cross any of them. The fixed obstacles are shown by black circles, and the linear polymer chain is shown by a black strand. The grey dashed lines and curves describe the polymer network. (**B**) Conceptualization of reptation of an infinitely long polymer chain based on the tube model proposed by Edwards and Doi. The polymer chain, shown by black strand, is confined in the tube (olive) of a certain diameter ‘a’ and allowed to move along the contour of the tube. The grey dashed lines and curves describe the polymer network. (**C**) Sticky reptation model of polymer entanglement as proposed by Leibler, Rubinstein, and Colby in the context of associative polymers possessing several ‘associating’ groups (stickers). Initial stage (on left): The linear chain P (black strand) has a crosslink I with chain P_1_ (dark gold strand). P_2_ (purple) represents the next available chain for crosslink formation. Final stage (on right): a new crosslink F is formed with another chain P_2_ (dark gold). In general, the chain that belongs to the crosslink is shown by dark gold strands with yellow circle representing the ‘closed stickers’, otherwise it is shown by purple. During this period, the center of mass of section CD of chain P is moved in a random manner. Details are explained in the main text.

Another intriguing topological concept, entanglement, emerged in the polymer physics field more than half a century ago, providing a new understanding of the physical properties of polymer melts and polymer motion in gels. The basic idea is that because polymer chains cannot cross through each other (without breaking bonds), under the above conditions, any polymer chain can be viewed as existing within a set of obstacles made up of all the other polymer chains surrounding it. A theory for this situation was developed by de Gennes for polymer motion in an environment of fixed obstacles such as crosslinked gels [39,61] and is illustrated in Figure 4A. Lateral motions of the polymer chain are, therefore, difficult because they are constrained by this crosslinked or entangled matrix of obstacles (in the original paper, the obstacles do not move). Thus, the polymer moves by a reptation motion, along the polymer ‘longitudinal’ directions. The model by de Gennes provided several predictions, including that the translational diffusion constant of the chain would scale as M^−2^ (very small for larger polymers; M is the polymer molecular weight; compare to a predicted M^−0.33^ scaling for a spherical particle following the Stokes–Einstein equation). Edwards and Doi developed the related tube model (Figure 4B), in which the dynamics of the polymer chain are restricted within a tube formed by the (mean field of the) surrounding entangled chains, as discussed above, and similarly resulting in a reptation motion [62,63,64]. The early papers by Edwards et al. also made interesting predictions, including that the viscosity of entangled polymer solutions should follow a M^3^ scaling law (M is the polymer molecular weight), which rapidly increases for longer polymers [63]. Later single-molecule studies directly visualized this type of reptation motion in concentrated solutions of DNA [65] and actin filaments [66]. These models and various subsequent extensions and theoretical advancements that included incorporation of sticker interactions provide a mechanistic basis for the understanding of many rheological and microscopic dynamical properties of such polymer systems [38,67,68]. Recent work has begun to discuss the relevance of these concepts for biomolecular condensates.

Several reports have noted the potential for entanglement effects to constrain the dynamics of long polymeric components of biomolecular condensates. One interesting example has been discussed in regard to the dynamics of the nucleolus by Riback et al. [69] Here, measurements of rRNA dynamics were used to infer an entangled network, with rRNA production at transcriptional sites and their cleavage/processing resulting in vectorial motion and facilitation of release of pre-ribosomal particles at the nucleolus periphery. Another interesting example has been discussed by Nguyen et al. in the context of nucleotide expansion repeat sequences, which are linked to diseases such as Huntington’s disease and ALS [70]. Here, coarse-grained computational simulations revealed that these sequences form dense networks in condensates, with expanded molecular conformations (predicted by Flory [49]) and with reptation-like slow dynamics. Another example has been discussed for the case of TIS granules. These granules consist of mesh-like condensates that have common surface area with the endoplasmic reticulum and are important for the trafficking of membrane proteins. Using in vivo and in vitro experiments, Ma et al. showed that a minimal model of RNA-binding protein and mRNAs with disordered regions can recapitulate the formation of such irregular structures, presumably with entanglement effects contributing to the overall morphology and dynamics [71]. 

A variation of such entanglement effects is the case in which intermolecular interactions are topologically enforced by the formation of interlinked closed geometries such as rings or loops, envisioned in the form of an ‘Olympic gel’ of interlinked rings by de Gennes [39]. An interesting biological example of such a condensate is represented in the thousands of interlinked DNA rings in the kinetoplast DNA of Leishmania tarentolae, for which dissociation can only be achieved by a bond-breakage process. In a related study, Michieletto et al. have shown how biochemical reactions that alter the topology of entangled fluids can result in complex patterns of time-dependent rheological properties of these soft materials [72,73]. 

Given the prevalence of long RNA/protein modules and the potential for transient looped structures in biomolecular condensates, it is likely that entanglement effects play substantial roles in many condensates and their biological functions.

## 4. Polymer Rheology and Biomolecular Condensation

A growing body of evidence suggests that biomolecular condensation occurs via thermodynamically reversible PS, resulting in droplets with liquid-like properties. Recently, several studies have shown that many protein and nucleic acid droplets exhibit viscoelastic behavior, which is a characteristic of non-Newtonian fluid as opposed to a fluid such as water, a Newtonian fluid [72,74,75,76]. Recently, Michieletto and Marenda discussed several possible reasons behind this complex non-trivial behavior of condensates, such as the aging of the fluid as an outcome of a local increase in protein concentration driven by liquid–liquid phase separation, bridging-induced phase separation (BIPS) and the effects of percolating network formation based on the sticker–spacer framework of associative polymer models, which may/may not lead to the formation of physical gel depending on the connectivity pattern and other parameters of the system [72]. The viscoelastic behavior of condensates and gels is believed to be biologically relevant and implicated in disease and functions [15,77,78].

To understand the viscoelasticity of condensates, it is crucial to discuss the theory of reversible networks that sets the cornerstone of the modern view of condensate rheology. Reversible polymer networks are viscoelastic, showing intermediate features between Newtonian fluids and Hookean solids. They show enhanced viscoelastic behavior compared to polymers that lack associative groups or stickers [38]. The rheological properties of a physically reversible network are attributed to two criteria [79,80,81,82]: (1) the extra macroscopic relaxation process due to the making and breaking of temporary junctions, sticker-based crosslinks; and (2) the microscopic lifetime of junction points/sticker–sticker crosslinks implicated in the slower rate of crosslink formation and destruction compared to thermal motion of the polymer chain/strand. The Rouse model and reptation theories pictured by de Gennes took a mean-field approach to describe the relaxation process of polymer chains [38,61,67,83,84,85]. According to them, if the relaxation timescale of chains of similar size is the same, the dynamical properties of a single chain can be explained by considering the neighboring chains as a frictional environment, whereas the reptation model considers the neighboring chains as a tube-like confinement [62,63,64,86]. Reptation dynamics is a snake-like diffusion of a chain along the length of the tube, and the relaxation time of the entangled polymer melt/gel is the time it takes to reptate out of the tube [40,61]. The simple reptation model is not valid near the gel point due to the presence of precursor chains of sol of different sizes and topologies and the unique dynamical feature of the gel matrix governed by the sticker–sticker interactions [79]. The scaling law developed by Rubinstein and Semenov appears to be more suitable for quantifying the change in linear viscoelasticity in connection with the degree of gelation [27,80]. When the gel network is fully formed without leaving any sol chain in the system, the mean-field approach of reptation model is sufficient to describe the viscoelasticity of the system. Leibler, Rubinstein, and Colby demonstrated a sticky reptation model for the dynamics of entangled networks possessing several temporary crosslinks [38]. Figure 4C depicts a fundamental process of chain diffusion in a reversible gel governed by sticker–sticker interactions, as proposed by Leibler, Rubinstein, and Colby. According to this model, a closed sticker that belongs to the crosslink I (yellow circle) between the chains P (black) and P_1_ (dark gold) is allowed to move distances of the order of the confining tube diameter. Therefore, crosslink I does not allow the diffusion of unentangled loops of the chain P between closed stickers C (yellow circle) and D (yellow circle). CI and DI, which are the parts of the chain P between the closed stickers, undergo Rouse-like motions with almost fixed ends, meaning their center of mass changes around their average positions [38]. When the crosslink I opens, the free sticker moves. If it is assumed that the equilibration time of the strand CD is shorter than the lifetime of the open sticker, and the sticker C and D remains closed within this timescale, the sticker would either recombine with chain P_1_ at the crosslink I, resulting in zero net displacements, or it would associate with a different chain P_2_ (purple), resulting in the formation of a new crosslink F (yellow circle) (Figure 4C) [38]. During the process from breaking crosslink I to making the crosslink F, the center of mass of the section of chain P moves to a new average position with the assumption that the stickers remain closed during the equilibration of the strand. Such displacement motion along the tube results in a reptation, such as the diffusion of the linear chain P. Overall, they suggested that if the relaxation timescale is shorter than the lifetime of the crosslink, the network exhibits elastic behavior, whereas the chain diffusion along the confining tube is governed by the sequential destruction of only a few crosslinks on a longer timescale.

Keeping the essence of the polymer rheology theories in mind, it is suggestive that the droplet-spanning percolated network with precise dynamical properties, the degree of crosslinking, relaxation due to dissociation and reassociation of stickers, and the microscopic lifetime of sticker-mediated crosslinks may contribute to the viscoelasticity and other material properties of the condensates. Crosstalk among soft matter physics, rheology, polymer physics, and fluid mechanics is necessary to elucidate the physical underpinning of condensate viscoelasticity.

## 5. Concluding Remarks and Future Directions

In recent years, the concept of network theory, in which the ‘links’ represent the interaction between the elements has gained significant importance in analyzing and predicting the behavior of complex biomolecular systems [87]. For example, protein–protein interactions networks are substantially implicated in cellular structure and function [88]. The revolution of network theory prompted the idea of the application of topology-based models to characterize a multitude of principal biological phenomena including biomolecular condensation, protein folding, gelation, and connectivity arrangements at molecular, cellular, and tissue scales in response to stress and ailments. Furthermore, there is increasing application of concepts from polymer physics to biological systems and materials. Along these lines, understanding the physical bases of percolation and entanglement in these systems is expected to be important for better definition of their links to biomolecular condensation. As discussed earlier, the application of percolation/entanglement concepts in this area is at relatively early stages and has current limitations as well. Correspondingly, substantial future work is needed (and expected) towards testing the applicability, generality and implications of these ideas.

Along these lines, the discoveries and concepts reviewed here may lay the groundwork for addressing a plethora of unexplored areas. These include more direct tests of percolation and entanglement approaches through the use of single-molecule or advanced rheology measurements [74,75,76] combined with molecular/cell biology and computational tools. It will also be important to carry out systematic studies to test the applicability and limitations of percolation and entanglement concepts, both in model and complex protein and RNA systems in vitro and in vivo. Additionally, more detailed studies of the distributions and dynamic properties of percolation clusters are needed, which can then allow more in-depth analysis using analytical theory and computational results. Here again, advanced single-molecule/particle and imaging methods will likely be particularly useful. The dynamical behavior of these clusters and, eventually, their conversion into the larger system-spanning droplets will be of great importance for investigation. As it accounts for the formation of connected clusters (or lattice animals, which essentially stands for a set of distinct connected clusters, also called animals, and which could be considered to be the equivalent of connected percolation clusters), the percolation approach could be useful in elucidating the formation of microgels, the first stage of the gelation process, with the spherical cross-linked microscopic network containing only finite clusters [89]. Other lines of future study include a more detailed mechanistic understanding of the physical underpinnings of sol–gel transitions coupled/decoupled with phase separation with the incorporation of different models, the interplay of protein/RNA conformational properties and complex/dynamic substructure in multicomponent and active-matter systems, better mapping of different types of sticker–spacer architecture in terms of percolation, and links to function [1,5,23,32,72,90,91,92] and the interplay/relevance of other mechanisms of cluster formation. Another captivating area to investigate is the condition where $C_{perc}<C_{sat}$, which essentially means that the system exceeds the connectivity threshold, thereby switching from dispersed monomers/clusters (sol) to the system spanning percolated matrix or physical gel without phase separation [23]. Physical gels are characterized by reversible noncovalent crosslinking. In the context of associative polymers with the sticker–spacer framework, if the bulk concentration of the interaction motifs is above the gel point but below $C_{sat}$, a connectivity transition occurs without droplet formation. We surmise that depending on the connectivity feature of the structures, the system will either form a physical gel or a distribution of network clusters with percolating behavior, but further investigation is necessary to elucidate the physical underpinnings of this shift in biological contexts. Gelation without phase separation is biologically relevant in many aspects [93,94,95,96]. Studies by Halfmann demonstrated that the phase transition of low-complexity sequence proteins to an amorphous solid or glass, a process based on the principle of vitrification, can be viewed as a phenomenon of gelation without phase separation [94]. The bacterial cytosol also exhibits sol-to-gel conversion akin to glass transition and impacts the mobility and fluidity of the cytoplasmic component in a size-dependent fashion [95,96]. Another interesting example is a report of analytical theory developed to understand the biology of actin networks, showing that actin-binding proteins that modulate connectivity can result in complex percolation-related behavior that can alter rheology and function [97]. Entanglement concepts are also being applied to explain several phenomena related to the diffusion and rheology of biomolecular condensates. Recent work by Nguyen et al. demonstrated that the mobility of RNA inside the highly viscous and dense droplets follows the reptation model of polymer entanglement [70]. Recently, Tom et al. demonstrated that at a relatively low concentration of Mg^2+^ induces short polyA-RNA sequences to form droplets that appear as internally arrested species [98]. They discovered that RNA chains exhibit slow translational dynamics, potentially with contributions from the entanglement effect within the densely packed RNA–RNA networking in the droplet state.

Because biomolecular condensation involves large, complex networking connectivity and intricate interactions between the interacting modules, it is a challenging task to quantify or decouple all these mechanistic aspects. We believe that the amalgamation of different models, techniques, and theories, along with the existing knowledge of percolation models, polymer entanglement, and phase-transition physics will further illuminate the inner workings of condensate science and their functions in biology.

## Data Availability

Not applicable.
